# Early measurement of IL-10 predicts the outcomes of patients with acute respiratory distress syndrome receiving extracorporeal membrane oxygenation

**DOI:** 10.1038/s41598-017-01225-1

**Published:** 2017-04-21

**Authors:** Chia-Hsiung Liu, Shuenn-Wen Kuo, Wen-Je Ko, Pi-Ru Tsai, Shu-Wei Wu, Chien-Heng Lai, Chih-Hsien Wang, Yih-Sharng Chen, Pei-Lung Chen, Tze-Tze Liu, Shu-Chien Huang, Tzuu-Shuh Jou

**Affiliations:** 1 Graduate Institute of Clinical Medicine, College of Medicine, National Taiwan University, Taipei, Taiwan; 2 Department of Surgery, National Taiwan University Hospital, Taipei, Taiwan; 3 Graduate Institute of Medical Genomics and Proteomics, College of Medicine, National Taiwan University, Taipei, Taiwan; 4 Department of Medical Genetics, National Taiwan University Hospital, Taipei, Taiwan; 5 Genome Research Center, National Yang-Ming University, Taipei, Taiwan

## Abstract

Patients diagnosed with acute respiratory distress syndrome are generally severely distressed and associated with high morbidity and mortality despite aggressive treatments such as extracorporeal membrane oxygenation (ECMO) support. To identify potential biomarker of predicting value for appropriate use of this intensive care resource, plasma interleukin-10 along with relevant inflammatory cytokines and immune cell populations were examined during the early and subsequent disease courses of 51 critically ill patients who received ECMO support. High interleukin-10 levels at the time of ECMO installation and during the first 6 hours after ECMO support of these patients stand as a promising biomarker associated with grave prognosis. The initial interleukin-10 level is correlated to other conventional risk evaluation scores as a predictive factor for survival, and furthermore, elevated interleukin-10 levels are also related to a delayed recovery of certain immune cell populations such as CD14^+^CD16^+^, CD14^+^TLR4^+^ monocytes, and T regulator cells. Genetically, high interleukin-10 is associated to two polymorphic nucleotides (−592 C and −819 C) at the interleukin-10 gene promoter area. Our finding provides prognostic and mechanistic information on the outcome of severely respiratory distressed patients, and potentially paves the strategy to develop new therapeutic modality based on the principles of precision medicine.

## Introduction

Acute respiratory distress syndrome (ARDS) is characterized by immense inflammatory lung injury, which is associated with high morbidity and mortality in intensive care unit (ICU)^1, 2^. Extracorporeal membrane oxygenation (ECMO) is an option for treating ARDS associated hypoxemia that is refractory to conventional ventilation^3, 4^; however, the beneficial role of ECMO in ARDS remain highly controversial^5, 6^. Therefore, identification of prognostic factors is a pivotal issue for appropriate use of this intensive care resource.

Given that the main causes of death in ARDS patients are multiple organ failure (MOF) and sepsis, presumably resulting from a systemic inflammatory response syndrome (SIRS), inflammatory cytokines interleukin (IL)-6, IL-8, IL-10, and immune cells such as T regulatory cell (Treg) have been hypothesized to predict the outcomes in ARDS patients^7–9^. However, their prognostic roles are completely unknown in more severe patients who need ECMO support. We have reported that plasma IL-10 possesses a predictive value for outcomes in patients with cardiogenic shock after ECMO intervention^10^. A continuous study with a larger cohort showed that cytokine storm is a hallmark in the non-survivors^11^, and the plasma IL-10 at 24 h after ECMO support can distinguish cardiogenic shock patients who succumbed from those who eventually survived to hospital discharge (Supplementary Figure S1).

IL-10 is a key immune-regulator during SIRS or infection with a variety of pathogens^12, 13^, which ameliorates possibly exaggerated pro-inflammatory responses. As delicately orchestrated immune response is crucial for a smooth resolution through SIRS, unbalanced pro- and anti-inflammation tilts the outcome toward mortality, either through outraged inflammatory responses or failure to protect against infectious organisms. The latter is caused by the persistence of a marked compensatory anti-inflammatory response syndrome (CARS) which is characterized by IL-10 over-production that suppresses tumor necrosis factor expression, decreases human leukocyte antigen molecules on monocytes, and reduces lymphocytes by means of apoptosis^14–16^. We thus hypothesize that IL-10 may have prognostic value in ARDS patients with ECMO treatment. To test this hypothesis, plasma IL-10, several inflammatory cytokines, and relevant immune cell populations were assessed in severe ARDS patients receiving ECMO support.

## Results

### Demographics and clinical characteristics of the patients

Fifty-one ARDS patients receiving ECMO support were prospectively enrolled in this study. Twenty of 21 ICU survivors survived to hospital discharge. Thirty patients died in ICU, and 24 of them could not be weaned from ECMO support. The baseline characteristics of these patients were shown in Table 1. Older age, lower BMI, and immunocompromised status were risk factors for ICU mortality. Conversely, patients afflicted with viral pneumonia had a more favorable outcome. Traditional evaluation systems, such as comorbidity index expressed as Charlson score, sequential organ failure assessment (SOFA), and acute physiology and chronic health evaluation (APACHE) II scores, all differentiated the death from the survival group. There were no significant differences in ventilator settings and rescue therapies between these two groups.

**Table 1:** Comparison of baseline characteristics before implementation with extracorporeal membrane oxygenation of the study subjects according to their survival status at ICU discharge.

Parameter	Survival (n = 21)	Death (n = 30)	*P* value
Age (years)	46.4 ± 14.3	59.5 ± 12.2	0.001
Male, *n* (%)	14 (66.7)	20 (66.7)	1.0
Body mass index (kg/m^2^)	26.8 (23.9~32.8)	24.3 (21.0~27.8)	0.043
Initial VA-ECMO, *n* (%)	3 (14.3)	5 (16.7)	1.0
ARDS diagnosis, *n* (%)			
Viral pneumonia	9 (42.9)	3 (10.0)	0.016
Bacteria pneumonia	3 (14.3)	7 (23.3)	0.495
Trauma	1 (4.8)	1 (3.3)	1.0
Aspiration pneumonitis	0 (0.0)	2 (6.7)	0.506
Extra-pulmonary sepsis	1 (4.8)	2 (6.7)	1.0
Autoimmune diseases	0 (0.0)	7 (23.3)	0.033
Postoperative	1 (4.8)	1 (3.3)	1.0
Other acute pneumonia	6 (28.6)	7 (23.3)	0.673
Ventilator setting			
PEEP (cmH2O)	13.6 ± 4.8	12.5 ± 3.8	0.346
MAP (mmHg)	21.4 ± 4.6	19.5 ± 5.3	0.183
Tidal volume (mL/kg)	5.5 (3.0~6.6)	6.3 (4.5~7.7)	0.064
PIP (cmH2O)	32.0 ± 7.0	31.0 ± 4.8	0.536
PaO2/FiO2	66.8 (52.0~74.9)	60.8 (52.6~89.1)	0.853
Duration of ventilation (days)	1.3 (0.3~2.0)	2.0 (0.9~7.9)	0.044
Rescue therapy, *n* (%)			
Bicarbonate	9 (42.9)	13 (43.3)	0.973
Nitric oxide	6 (28.6)	11 (36.7)	0.546
Neuromuscular blocker	16 (76.2)	20 (66.7)	0.463
Steroid	2 (9.5)	8 (26.7)	0.167
Pre-ECMO condition, *n* (%)			
Hypertension	9 (42.9)	10 (33.3)	0.489
Diabetes mellitus	6 (28.6)	8 (26.7)	0.881
Renal dialysis	3 (14.3)	7 (23.3)	0.495
Immunocompromised^a^	3 (14.3)	13 (43.3)	0.035
Charlson score	2 (1~4)	4 (3~8)	0.007
SOFA score	9 ± 3	14 ± 5	<0.001
APACHE II score	13 ± 5	22 ± 9	<0.001
RESP score	2 (1~4)	−1 (−3~0)	<0.001

Continuous data values are shown as medians with inter-quartile ranges for variables with non-normally distributed characteristic, or means ± standard deviation for variables following normal distribution pattern. The number of patients with frequency (percentage, %) is shown for categorical data. The listed *P* values of statistical tests were calculated using Mann–Whitney U or Student’s t test for continuous data and the *χ* ^*2*^ or Fisher’s exact test for categorical data. ^a^Immunocompromised is defined as hematological malignancies, solid tumor, solid organ transplantation and/or cirrhosis. VA-ECMO, venous-arterial extracorporeal membrane oxygenation; ARDS, acute respiratory distress syndrome; PEEP, positive end-expiratory pressure; MAP, mean airway pressure; PIP, peak inspiratory pressure; PaO_2_, partial pressure of oxygen; FiO_2_, fraction of inspired oxygen; SOFA, sequential organ failure assessment; APACHE, acute physiology and chronic health evaluation; RESP, respiratory extracorporeal membrane oxygenation survival prediction.

### Early elevation of IL-10 predicts clinical outcomes

**Figure 1:**
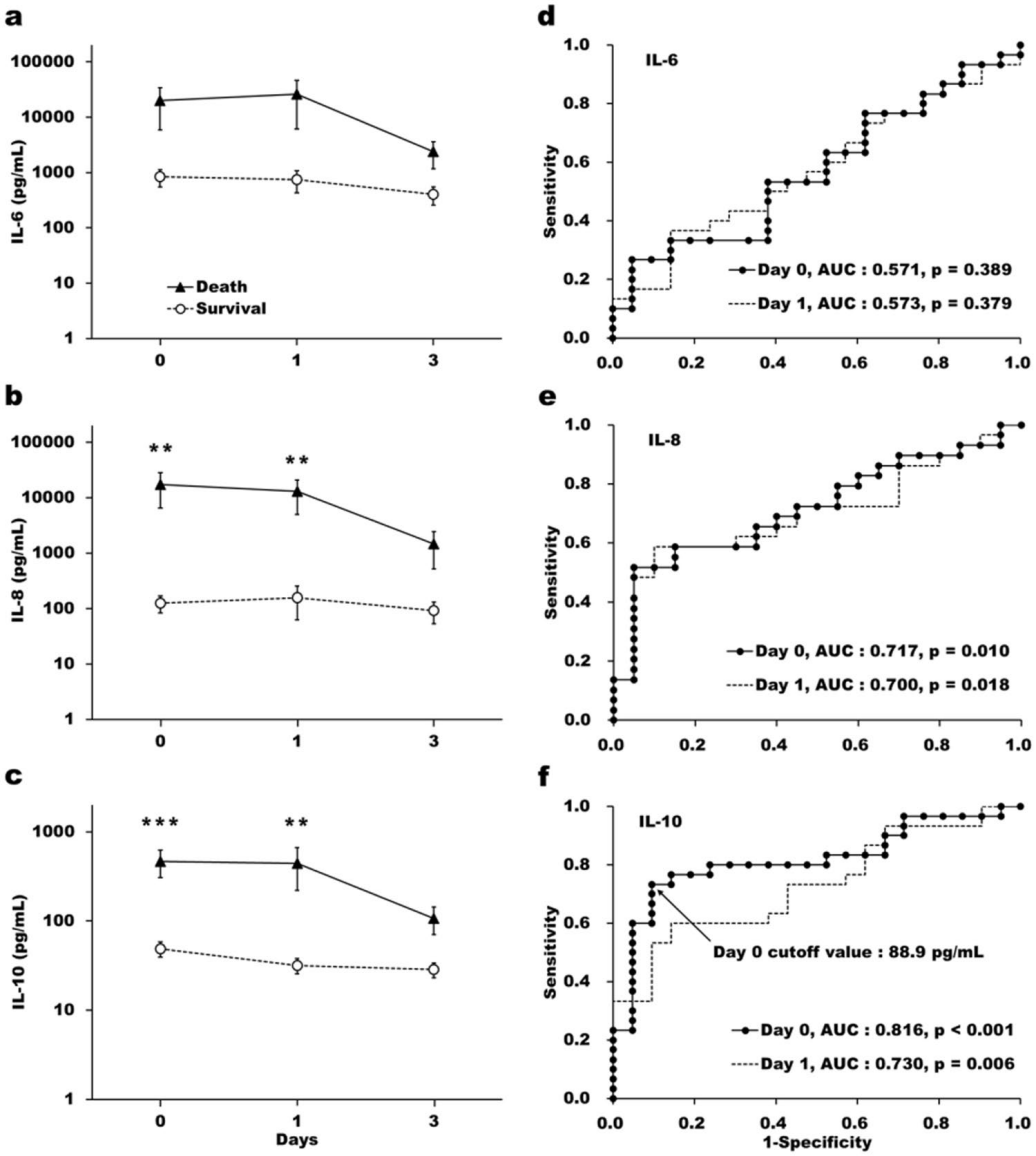
Initial plasma interleukin-10 level was a better prognostic biomarker than interleukins 6 and 8 in ARDS patients receiving ECMO support. Plasma interleukin-6 (**a**), interleukin-8 (**b**), interleukin-10 (**c**) concentrations were assessed in survivors (n = 21, *open circles*) and non-survivors (n = 30, *solid triangles*) at 0, 1, and 3 days after receiving ECMO support. Receiver-operating characteristic (ROC) analysis showed dissimilar predictive capabilities among initial cytokine values for ICU mortality. (**d**) Interleukin-6 levels at neither day 0 nor day 1 after ECMO support could differentiate the survivors from the non-survivors. Both Interleukin-8 (**e**) and interleukin-10 (**f**) predicted the outcome well by analysis using the ROC curve at day 0 and 1. However, the best predictive ability was tested in interleukin-10 level at day 0, with the optimal cutoff value at 88.9 pg/mL. The data represented the means and standard errors of each group. Values were logarithmically transformed before bivariate comparisons. **, and *** stand for *P* < 0.01, and *P* < 0.001, respectively, between death and survival groups.

**Figure 2:**
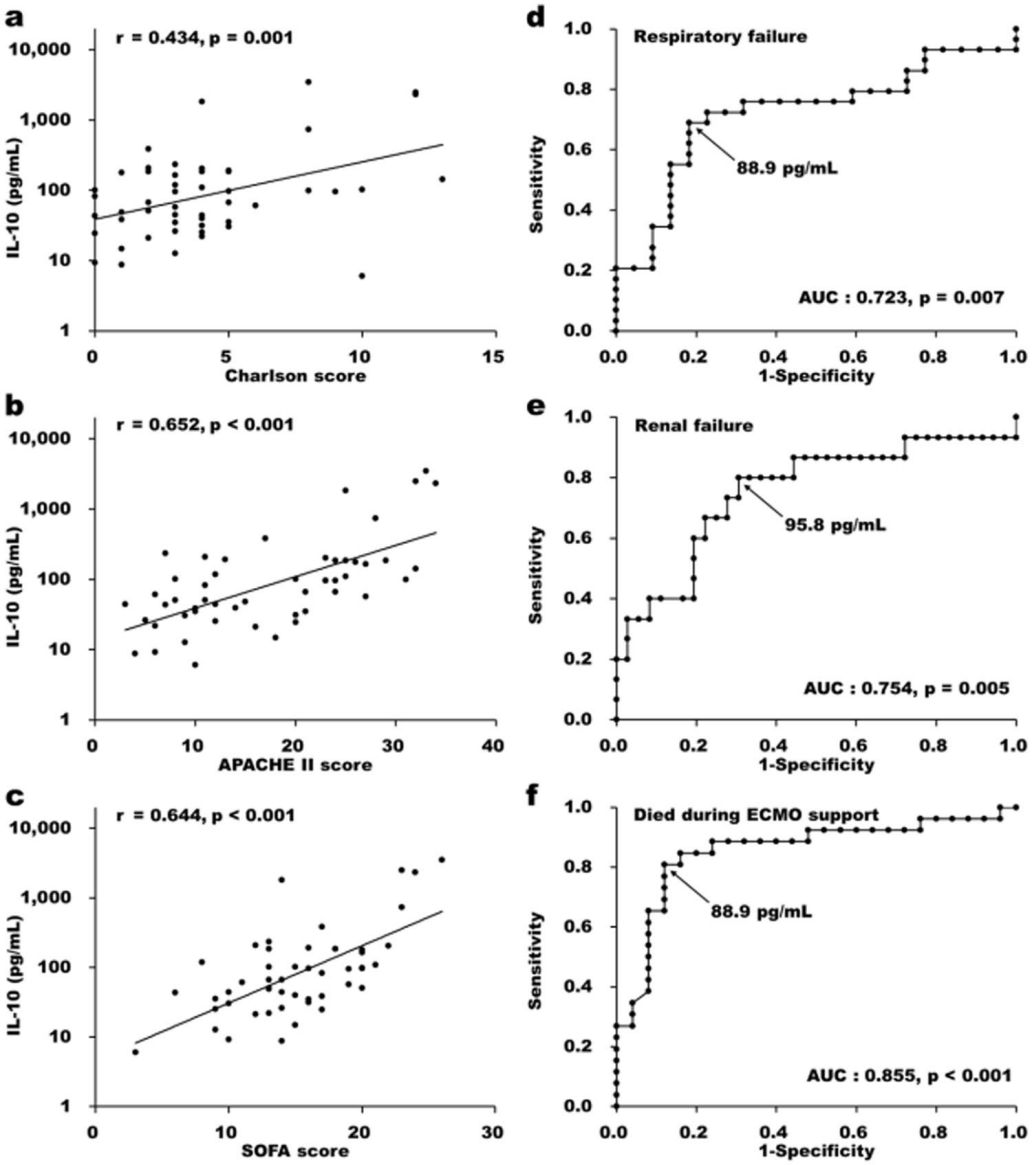
Initial plasma interleukin-10 levels were associated with higher chances of organ failures and mortality in ARDS patients during ECMO support. Plasma interleukin-10 level at day 0 was associated with the severity of illness as evaluated one day after receiving ECMO by comorbidity index Charlson (**a**), acute physiology and chronic health evaluation (APACHE) II (**b**), and combining sequential organ failure assessment (SOFA) score (**c**). Furthermore, Interleukin-10 level at day 0 predicted respiratory (**d**) and renal (**e**) failures in the death group, and served as an excellent predictive value for mortality during ECMO support (**f**), with an area under the receiver operating characteristic curve equaled 0.855.

Plasma cytokines were prominently higher in the death group at day 0 compared to the survival patients, especially for IL-8 and IL-10. The difference in these interleukin levels between survival and death groups diminished by day 3 after ECMO support (Fig. 1a,b and c). Although both the IL-8 and IL-10 concentrations were remarkably higher in the non-survivors than in survivors within one day after implementation of ECMO support, the best predictive ability for ICU mortality was tested in IL-10 level at day 0 with the area under the ROC curve (AUC) = 0.816 (Fig. 1d,e and f). Plasma IL-10 levels correlated well to both the Charlson comorbidity and APACHE scores (Fig. 2a and b). Similarly, the positive correlation between IL-10 level on day 0 and SOFA score on day 1 (Fig. 2c) denotes the instrumental role of plasma IL-10 in the development of multiple organ dysfunctions. Indeed, high plasma IL-10 levels correlated with the presence of respiratory and renal failures in our cohort (Fig. 2d and e). Furthermore, early IL-10 level can distinguish well between patients who died despite ECMO support and those who could be eventually weaned from this advanced life support (Fig. 2f).

### Tregs, CD14^+^CD16^+^, and CD14^+^TLR4^+^ cell populations were higher in survivors than non-survivors on day 3

**Figure 3:**
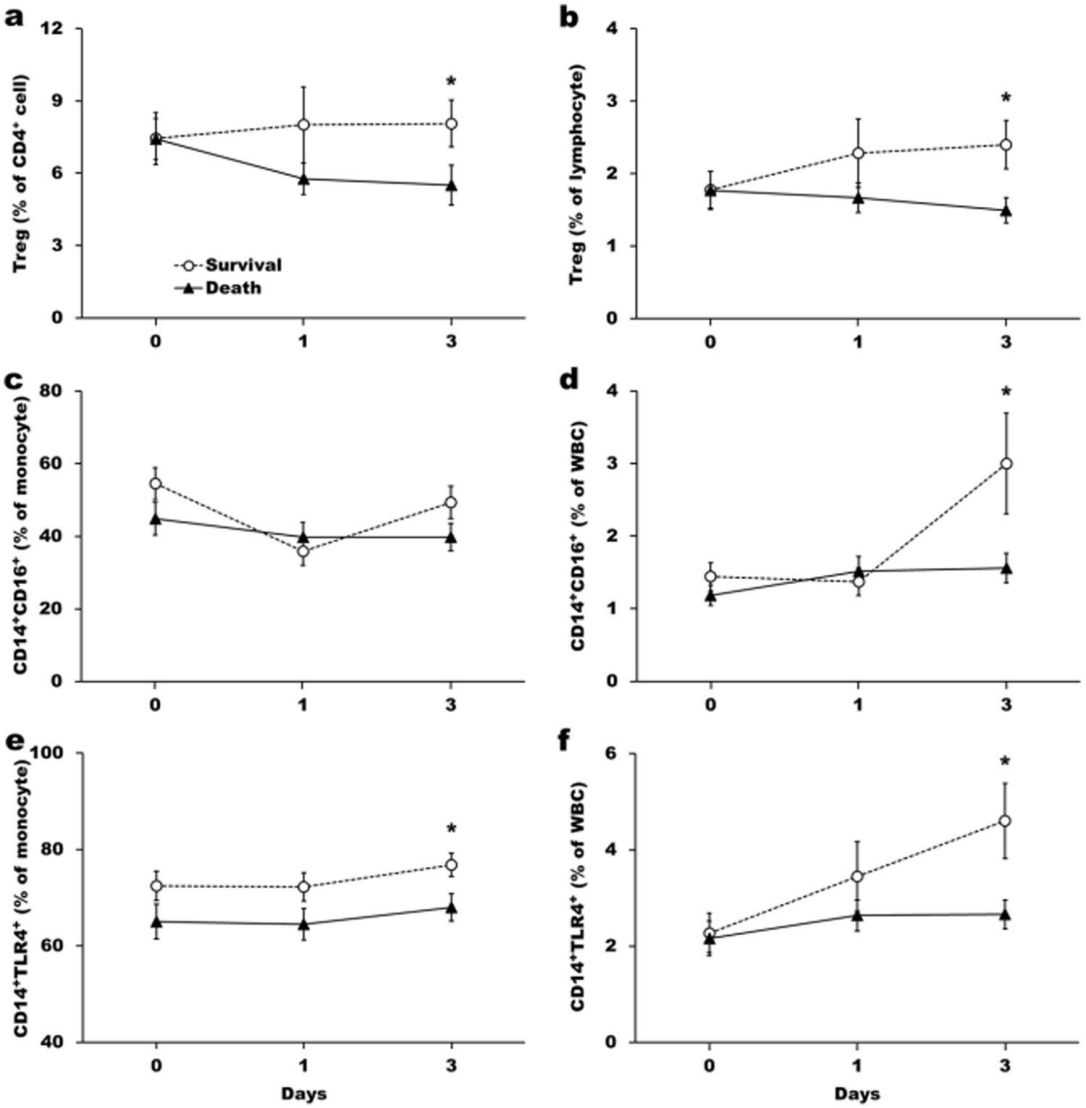
Comparisons of immune cell profiles between survivors and non-survivors in ARDS patients who received ECMO support. T regulator cell percentage in CD4^+^ lymphocytes (**a**), in total lymphocytes (**b**), CD14^+^CD16^+^ cell percentage in monocytes (**c**), in total white blood cells (**d**), CD14^+^TLR4^+^ cell percentage in monocyte (**e**), and in total white blood cells (**f**) were analyzed at day 0, 1 and 3 during ECMO support and compared between the survival and death groups. The data represented the means and standard errors of each group. Values were logarithmically transformed before bivariate comparisons. *Represents *P* < 0.05 between death and survival groups.

As cellular immune response is intimately involved in the regulations of cytokine production targeted against infection or inflammation, we also investigated the evolution of various lymphocyte and monocyte subpopulations during the early stage of ECMO support. CD3^+^, CD3^+^CD4^+^, CD3^+^CD8^+^ and CD19^+^ cells present in either total lymphocyte or WBC were not significantly different between survivors and non-survivors (Supplementary Figure S2). The percentages of some IL-10-producing immune cells differed substantially between survivors and non-survivors on day 3 after ECMO support. Among them, Treg percentages in CD4^+^ and total lymphocytes (Fig. 3a and b) were significantly lower in non-survivors than survivors on day 3. Although CD14^+^CD16^+^ cell percentages in monocyte were not different between these two groups (Fig. 3c), the CD14^+^CD16^+^ cell proportion of total white blood cells increased to a notably higher level in the survival than the death group on day 3 (Fig. 3d). Considerably higher CD14^+^TLR4^+^ proportions in monocyte (Fig. 3e) and total white blood cells (Fig. 3f) were also observed in the survivors than non-survivors on day 3.

### IL-10 level is an independent risk factor for ICU mortality

Those factors found significantly related to ICU mortality by univariate analysis (*p* < 0.05) were subjected to a logistic regression analysis. The result of multivariate logistic analysis indicated that initial plasma IL-10 level, age, and viral pneumonia are independently associated with ICU mortality (Table 2). Autoimmune disease was not introduced into the multivariate analysis because the patients with this etiology overlapped those with immunocompromised status. Furthermore, when those 7 patients with autoimmune disease were excluded from our analysis, the results of ROC analysis for IL-10 did not change significantly (AUC = 0.807, *p* < 0.001). IL-10 is implicated in the regulation of a diverse cell types involved in innate and adaptive immune response. To elucidate the potential pathogenic role of IL-10 expressions in their ICU mortality, ARDS patients were categorized by their initial IL-10 levels according to the cutoff value noted in Fig. 1. The dynamic changes in the percentages of Treg, CD14^+^CD16^+^, and CD14^+^TLR4^+^ cells in the study subjects during the ECMO support period were examined (Supplementary Figure S3). Intriguingly, the dynamic profiles of these immune cells between the subjects categorized as survival and non-survival groups (Fig. 3) were very similar to those categorized according to initial IL-10 levels (Supplementary Figure S3). This result implies that IL-10 plays a pivotal role in modulating the differentiation of specific immune cells in ARDS patients and significantly contributes to the outcomes of ARDS patients after ECMO intervention.

**Table 2:** Univariate and multivariate logistic regression analyses for independent predictors of ICU mortality in the patients of this study.

Variables	Univariate		Multivariate		
	OR	*P*	OR	95% CI	*P*
IL-10 ≥ 88.9 pg/mL	26.125	<0.001	51.531	2.798~948.940	0.008
Age ≥ 59.5 years	8.500	0.002	22.234	1.760~280.831	0.017
Viral pneumonia	0.148	0.011	0.024	0.001~0.568	0.021
Immunocompromised^a^	4.588	0.035	4.332	0.297~63.221	0.284
Body mass index, kg/m^2^	0.898	0.053	1.052	0.875~1.265	0.588
Pre-ECMO ventilator days	1.240	0.050	1.538	0.935~2.531	0.090

OR, odds ratio; CI, confidence interval. ^a^Defined as hematological malignancies, solid tumor, solid organ transplantation and/or cirrhosis.

### Predictive accuracies of IL-10 level compared to other risk scoring systems for ICU mortality for this study cohort

**Figure 4:**
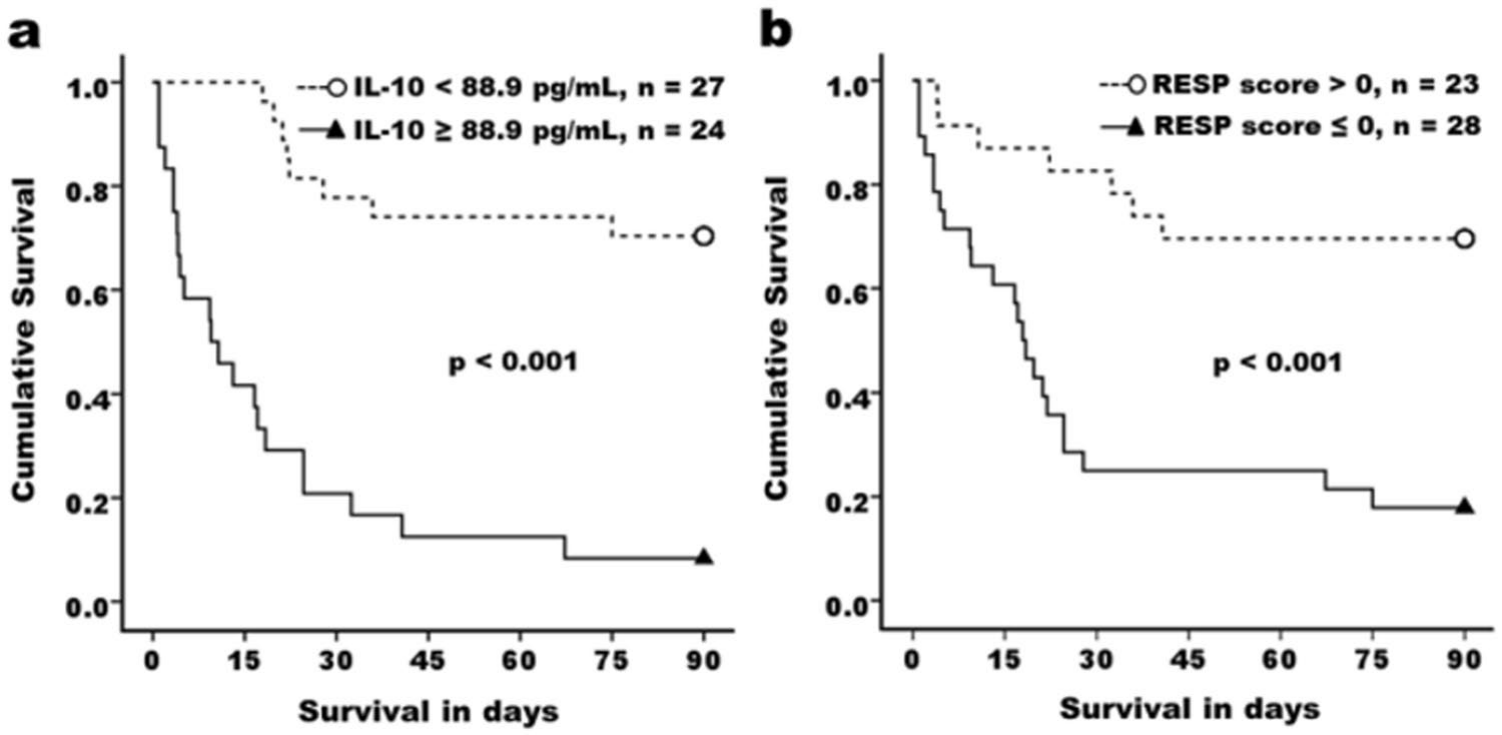
Kaplan–Meier analysis for 90-day survival probability in ARDS patients receiving ECMO support according to initial IL-10 levels and RESP scores. ARDS patients with higher plasma IL-10 levels at day 0 (**a**) and lower RESP scores (**b**) before ECMO implementation were associated with significantly worse survival.

The initial IL-10 level predicted ICU mortality well with specificity and positive predictive value of 90.5% and 91.7%, respectively (Supplementary Table S1). This attribute makes IL-10 a legitimate biomarker of predictive power comparable to other conventional ICU scoring systems, such as SOFA and APACHE scores. RESP score is a recently developed evaluation system to predict the prognosis of ARDS patients after ECMO support. Compared to RESP system, which combines twelve clinical and laboratorial assessments, initial IL-10 value apparently possessed a non-inferior predictive power. Early IL-10 assessment presented a better specificity (90.5 vs 76.7%), positive predictive value (91.7 vs 82.1%), and negative predictive value (70.4 vs 69.6%), while RESP system was better than single IL-10 measurement in term of sensitivity (76.2 vs 73.3%) (Supplementary Table S1). Furthermore, Kaplan-Meier analysis demonstrated that patients with initial IL-10 levels higher than the optimal cut-off point of 88.9 pg/mL had a significantly higher hospital mortality rate (P < 0.001, Fig. 4a). The RESP score was in conformity with mortality prediction with those patients of RESP score less than 0, an optimal cut-off value set after AUC analysis (Supplementary Table S1), having a higher ICU mortality (P < 0.001, Fig. 4b).

### IL-10 promotor variants are associated with the initial IL-10 levels and clinical outcomes

**Figure 5:**
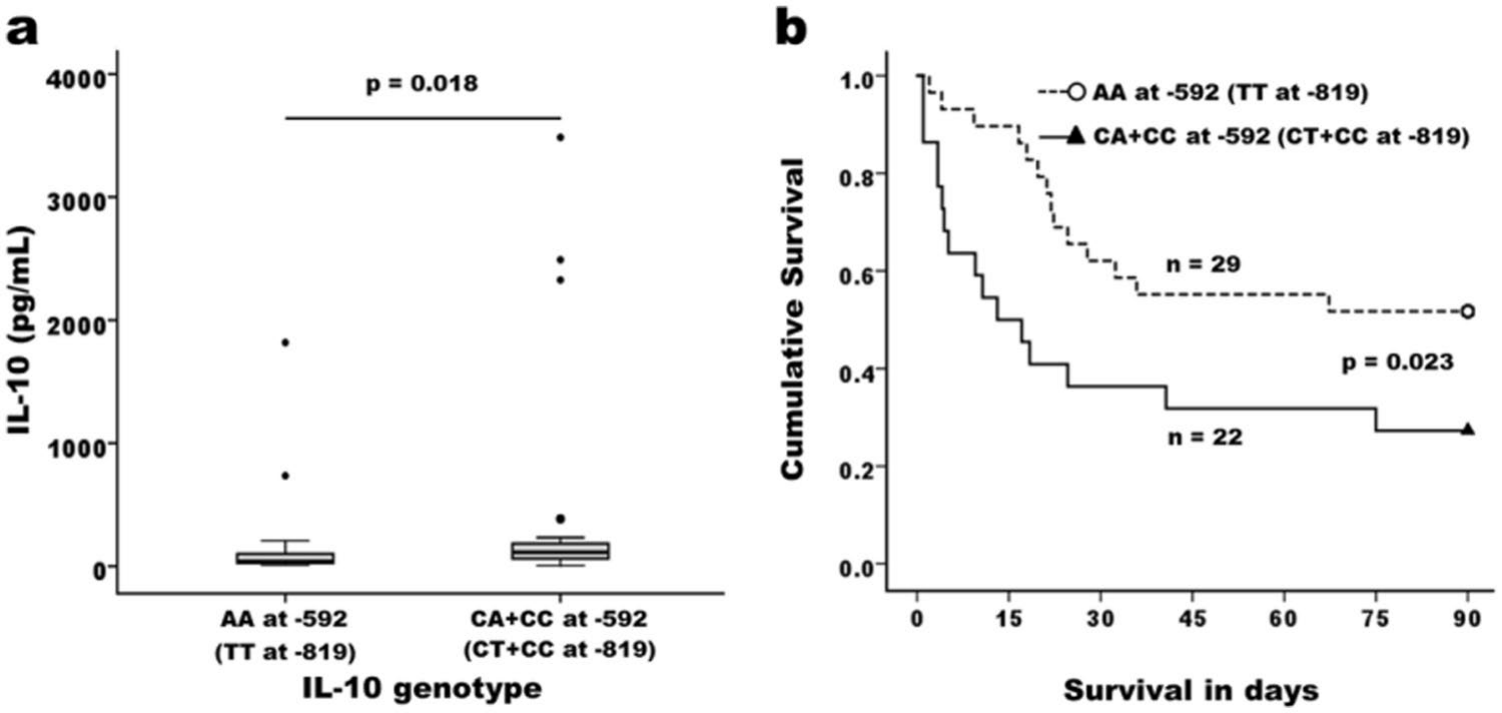
Effect of IL-10 genotypes on the initial plasma IL-10 concentrations and the 90-day survival probability after receiving ECMO support. ARDS patients with −592 AC and CC (−819 TC and CC) genotypes were associated with higher plasma IL-10 level (**a**) and had significantly worse survival after ECMO support (**b**).

Given the apparent difference present in the plasma IL-10 levels between survivors and non-survivors, we hypothesized that genetic variation may influence the extent of IL-10 production and secretion during the early phase of ARDS evolution. Three single nucleotide polymorphisms (SNPs) in the *IL-10* promotor: A-1082G (rs1800896), T-819C (rs1800871), and A-592C (rs1800872) have been reported to be associated with an increased or decreased of IL-10 production^17, 18^. Indeed, genetic association study revealed that the −819 C and −592 C alleles were observed with significantly higher frequencies in the patients with initial plasma IL-10 level ≥ 88.9 pg/mL (Table 3). In contrast, the TT and AA homozygotes frequencies of the −819 and −592 SNPs respectively, were increased in patients with lower initial plasma IL-10 levels. There was no difference in allele or genotype frequencies at the −1082 locus. Not only correlated to the IL-10 levels, the variants at *IL-10* promoter were noted to be associated with the eventual possibility of weaning successfulness from ECMO in the study group (Supplementary Table S2). Those C allele carriers at −819 and −592 positions (the alleles at these two genomic loci are linked as discussed later) were associated with higher IL-10 level (Fig. 5a) and higher 90-day mortality (Fig. 5b) than the patients carrying non-C allele. Taken together, these results signify the biological basis of the pathogenic effect of IL-10 on the clinical outcome of ARDS victims undergoing severe inflammatory distress.

**Table 3:** Allele frequency and genotype distribution of the *IL-10* promotor variants in the study subjects according to their plasma IL-10 levels at day 0.

Allele/genotype	Low IL-10 (n = 27)	High IL-10 (n = 24)	*P* value	OR (95% CI)
Allele, n (%)				
−1082 A	53 (98.1)	44 (91.7)	0.185	N.S.
−1082 G	1 (1.9)	4 (8.3)		N.S.
−819 T	47 (87.0)	28 (58.3)	0.001	0.209 (0.078~0.555)
−819 C	7 (13.0)	20 (41.7)		4.796 (1.801~12.774)
−592 A	47 (87.0)	28 (58.3)	0.001	0.209 (0.078~0.555)
−592 C	7 (13.0)	20 (41.7)		4.796 (1.801~12.774)
Genotype, n (%)				
−1082AA	26 (96.3)	21 (87.5)	0.429	N.S.
−1082AG	1 (3.7)	2 (8.3)		N.S.
−1082GG	0 (0.0)	1 (4.2)		N.S.
−819TT	20 (74.1)	9 (37.5)	0.008	0.210 (0.064~0.693)
−819TC	7 (25.9)	10 (41.7)		N.S.
−819CC	0 (0.0)	5 (20.8)		N.S.
−592AA	20 (74.1)	9 (37.5)	0.008	0.210 (0.064~0.693)
−592AC	7 (25.9)	10 (41.7)		N.S.
−592CC	0 (0.0)	5 (20.8)		N.S.

All the *P* values represent *χ* ^2^ or Fisher’s exact test results. OR, odds ratio; CI, confidence interval. N.S., non-significant.

## Discussion

We discovered that plasma IL-10 levels in ARDS patients were correlated with severity of illness during ECMO institution. The early increased IL-10 predicted unsuccessful ECMO weaning in the ensuing period of ICU stay and eventual mortality. This characteristic of IL-10 endured even after adjusting for various confounding factors such as age, etiology of ARDS, immunocompetence, BMI, and duration of mechanical ventilation before ECMO institution (Table 2). The initial IL-10 levels were linked to the genetic variations in the gene promotor region of this cytokine.

IL-10 can be produced by many different myeloid and lymphoid cells, including CD14^+^ CD16^+^ cells and Tregs respectively^19–22^. TLR4, together with its co-receptor CD14, plays a central role in innate immunity by initiating a signaling cascade for inflammation after engagement with lipopolysaccharide (LPS) or tissue damage associated molecule patterns (DAMPs)^23–25^. Patients with lower TLR4/CD14 expression on their monocytes have been shown to more likely succumb to sepsis^26, 27^. The persistently fewer CD14^+^CD16^+^ and CD14^+^TLR4^+^ monocytes in the non-survivors of ARDS could be caused by intensive endocytosis of CD14/TLR4 due to rampant DAMPs. The recovery of these receptors on cellular surface may depend not only on the clearances of these pathogens or danger molecules, but also on the initial plasma IL-10 level which stimulates endocytic activity of monocytes^28^. Furthermore, there are evidences for a reciprocally regulatory relationship between IL-10-producing cells and IL-10^12, 29, 30^. Thus, higher plasma IL-10 in the non-survivors of ARDS indicates that an immunoparalysis status exists in these cases. This might explain why excessive IL-10 production could relate to higher SOFA score, and lead to malfunction of multiple organs and poor outcomes.

Although a number of prognostic algorithms have been recently advocated for ARDS patients requiring ECMO^31–34^, these evaluation modalities generally incorporate numerous factors that construct a universally applicable risk-prediction system. Whereas, limited use or absent registration of one or more factors related to outcome makes the application of these scoring systems restrained in some centers, and the predictive performances may be nullified by those determinants due to a wide spectrum of patient sources^35–37^. Thus, simple criteria that can be followed conveniently would be more bedside practical. Recently, Roch *et al*. reported that age, influenza pneumonia, and SOFA score are independent factors significantly related to hospital mortality in a cohort of 85 ARDS patients equipped with ECMO, and a simple scoring system based on these three factors was constructed^32^. Their finding is quite similar to ours taking into account that the initial plasma IL-10 level is highly correlated to SOFA score in our patients. However, it is worth noting that the optimal age derived from ROC analysis to differentiate the outcome is different between these two cohorts (45 vs. 59.5). This may be due to different patient sources and indications for implementing ECMO, highlighting the unstable nature of single factor in outcome prediction. Accordingly, the optimal IL-10 level to distinguish ICU mortality may vary in other centers.

Our finding that two high IL-10 producing genetic variants (−819C and −592C) is associated with poor outcome in severe ARDS cases receiving ECMO is contradictive to an earlier report which demonstrates that the high IL-10 producing −1082GG genotype is protective against mortality and organ failure in ARDS^38^. It is unknown at present why there exists such a discrepancy, but racial difference could be one of the reasons. In contrast to other western ethnic groups, who have a more variable allelic distribution at the −1082 locus of *IL-10* gene^38, 39^, there is a biased higher frequency of “A” alleles (97 out of 102 in total 51 study subjects, Table 3) in our patients. Another point worth mentioning is that there is a haplotype linkage between the −819 “T” allele to the −592 “A” allele and −819 “C” allele to the −592 “C” allele, respectively, in our study subjects (Table 3). The higher frequency of −1082A and the genetic linkage of −819 and −592 alleles have been observed in many studies which recruit ethnic groups similar to ours^40–42^. ARDS is a complex and heterogeneous syndrome that involves multiple pathogenic pathways and affects a diverse spectrum of patients who often have comorbid illnesses^43^, hence it is difficult to ascertain the influence of genetic heterogeneity on clinical outcomes in ARDS. Considerably additional researches are necessary to understand the impact of the *IL-10* gene variants contributing to alterations in ARDS outcomes.

## Methods

### Study population and data collection

Adult ARDS patients admitted to the ICU of National Taiwan University Hospital for ECMO support were prospectively enrolled between October 2011 and April 2016. The indication of venovenous-ECMO is a ratio of partial arterial pressure of oxygen/fraction of inspired oxygen (PaO2/FiO2) <80 mmHg under positive end-expiratory pressure of at least 5 cmH_2_O. Venoarterial-ECMO is indicated if significant pulmonary hypertension, cardiac dysfunction associated with sepsis, and arrhythmia became apparent. Blood samples were withdrawn from patients before ECMO installation (0 h), 2, 6, 24, and 72 h after oxygenation for cytokines and flow cytometric analysis. There were fifteen study subjects whose specimens could not be timely collected before ECMO installation. For these cases, the first samples collected either at 2 h (n = 8) or 6 h (n = 7) would be processed instead with the result of assays performed on samples collected at 0~6 h defined as day 0 data in our subsequent analyses. The primary outcome of this study was death in ICU, and other outcomes including death during ECMO support and specific organ failure associated with mortality were also recorded. The protocol for blood sampling was approved by the Institutional Review Board (IRB) (IRB number 201103056RB), and it was performed according to the principles of the Declaration of Helsinki. Written informed consents were obtained from the closest relatives of every recruited patient. Vital demographics and clinical variables for each patient were also collected according to another approved study protocol (IRB number 201002034 R), by which the informed consents were waived.

### Cytokine and chemokine analysis

Blood from the recruited patients was withdrawn into ethylenediaminetetraacetic acid (EDTA) containing tubes (Vacutainer, Becton–Dickinson, San Jose, CA), kept on ice, and centrifuged at 2000*g* for 20 min at 4 °C to separate plasma, which were aliquoted and stored at −80 °C until analysis. Interleukin (IL)-6, IL-8, and IL-10 levels were measured by the commercial enzyme-linked immunosorbent assay (ELISA) kits (BD Biosciences) according to the manufacturer’s instructions.

### Flow Cytometric analysis

One hundred microliters of EDTA anticoagulated whole blood was mixed with the combination of the following mouse anti-human antibodies (BD Biosciences, San Jose, CA) in three separate tubes. (1) 10 μL of peridinin chlorophyll (PerCP) conjugated anti-CD3, 10 μL of fluorescein isothiocyanate (FITC) conjugated anti-CD4, 10 μL of phycoerythrin (PE) conjugated anti-CD8, and 10 μL of allophycocyanin (APC) conjugated anti-CD19. (2) 12 μL of APC conjugated anti-CD25, 10 μL of PerCP conjugated anti-CD3, 10 μL of FITC conjugated anti-CD4, and 2 μL of PE conjugated anti-CD127. (3) 10 μL of PE conjugated anti-CD14, 10 μL of FITC conjugated anti-CD16, and 12 μL of biotinylated anti-toll like receptor 4 (TLR4) antibodies. After incubation for 20 min at room temperature in the dark, red blood cells were lysed by 1.5 mL of BD lysing buffer, and white blood cells were washed twice with 1.5 mL of PBS containing 1% fetal bovine serum and 0.1% sodium azide (washing buffer). After centrifugation, the cells in tube (1) and (2) were fixed in 0.5 mL of PBS with 0.25% paraformaldehyde (fixation buffer) and kept on 4 °C until analysis. The cells in tube (3) after first washing were further incubated with 5 μL of APC conjugated streptavidin (BD Biosciences, San Jose, CA) for another 20 min, followed by washing twice in washing buffer, and fixing in fixation buffer as those in tube (1) and (2). For each test, a minimum of 20,000 leukocytes were acquired on BD Calibur flow cytometer and analyzed with CellQuest software version 3.2. Neutrophils, lymphocytes, and monocytes were identified based on their forward and side scattered (FSC/SSC) light patterns by flow cytometry. CD4^+^CD25^+^CD127^low^ Tregs were analyzed using CD25 and CD127 markers on CD4^+^ gated population of T cells.

### Genotyping for genetic variants

Genomic DNA in all the patients were extracted and purified from the peripheral blood leukocytes using the DNeasy Blood & Tissue kit according to the manufacturer’s instructions (QIAGEN GmbH, Germany). A set of primer pairs was designed with Primer3^44^ to amplify the 2 kb promoter region of *IL-10* gene. The PCR products were purified by ExoSAP-IT (GE Healthcare, USA) following by sequencing reactions using the BigDye Terminator v3.1 Cycle Sequencing Kit (Thermo Fisher Scientific Inc./Applied Biosystems, USA). The reaction products were purified and run on a 3730xl DNA Analyzer (Applied Biosystems). Variations of the *IL-10* gene were detected by Geneious version 8.0.5 (www.geneious.com)^45^.

### Statistical analysis

The data were analyzed using SPSS 17.0 (SPSS Inc, Chicago, IL). Categorical variables were presented as numbers percentages, and compared using *Χ* ^2^ or Fisher’s exact test. Continuous variables were assessed by Shapiro-Wilk test for normality of data distributions, and the significant differences between groups were compared by Mann–Whitney U test or Student’s *t* test. Plasma cytokine concentrations and immune cell percentages were presented as means with standard error of mean (SEM) in figures. Non-normally distributed data were logarithmically transformed before bivariate comparisons. The odds ratios (OR) and 95% confidence intervals (CI) were calculated using logistic regression. The correlated data were scattered as dotted plots and analyzed by the Pearson’s test. Receiver-operating characteristic (ROC) analysis was performed to validate the predictive ability for various outcomes and determine the optimal cut-off values by Youden index. The Kaplan–Meier survival curve was presented to show the survival differences between groups, and the log-rank test was used to calculate the statistical significance.

## Electronic supplementary material

Supplementary Materials
