# Supplementary material for: Early measurement of IL-10 predicts the outcomes of patients with acute respiratory distress syndrome receiving extracorporeal membrane oxygenation
Source: Sci Rep. 2017 Apr 21;7:1021. doi: 10.1038/s41598-017-01225-1 (PMC5430643; doi:10.1038/s41598-017-01225-1)
Supplement: Supplementary file 1 — Supplementary Materials [file 41598_2017_1225_MOESM1_ESM.pdf]

Supplementary Material

Early measurement of IL-10 predicts the outcomes of patients with acute respiratory distress syndrome receiving extracorporeal membrane oxygenation

Chia-Hsiung Liu [1, 2], Shuenn-Wen Kuo [2], Wen-Je Ko [2], Pi-Ru Tsai [2], Shu-Wei Wu[2], Chien-Heng Lai [2], Chih-Hsien Wang [2], Yih-Sharng Chen [2], Pei-Lung Chen [1, 3, 4], Tze-Tze Liu [5], Shu-Chien Huang [2*], Tzuu-Shuh Jou [1*],

1 Graduate Institute of Clinical Medicine, College of Medicine, National Taiwan University, Taipei, Taiwan;

2 Department of Surgery, National Taiwan University Hospital, Taipei, Taiwan;

3 Graduate Institute of Medical Genomics and Proteomics, College of Medicine, National Taiwan University, Taipei, Taiwan;

4 Department of Medical Genetics, National Taiwan University Hospital, Taipei, Taiwan;

5 Genome Research Center, National Yang-Ming University, Taipei, Taiwan.

* Corresponding authors: Shu-Chien Huang dtsurg99@yahoo.com.tw, and Tzuu-Shuh Jou jouts@ntu.edu.tw

Table S1. Comparison of the predictive values of IL-10 levels and other risk-evaluation scores for ICU mortality in the study subjects.

Factors	AUC	Cutoff value	Sensitivity, %	Specificity,%	PPV, %	NPV,%
IL-10 (pg/mL)	0.816	≥ 88.9	73.3	90.5	91.7	70.4
SOFA score	0.793	≥ 13	62.1	95.2	94.4	62.5
APACHE score	0.787	≥ 17	73.3	81.0	84.6	68.0
RESP score	0.813	≤ 0	76.2	76.7	82.1	69.6

AUC, area under the receiver-operating characteristic curve; PPV, positive predictive value; NPV, negative predictive value; IL, interleukin; SOFA, sequential organ failure assessment; APACHE, acute physiology and chronic health evaluation; RESP, respiratory extracorporeal membrane oxygenation survival prediction.

Table S2. Allele frequency and genotype distribution of the *IL-10* promotor variants in the study subjects according to their status at the moment of ECMO removal.

Allele/genotype	Weaned (n=25)	Not weaned (n=26)	*P* value	OR (95% CI)
Allele, n (%)				
–1082A	49 (98.0)	48 (92.3)	0.363	N.S.
–1082G	1 (2.0)	4 (7.7)		N.S.
–819T	43 (86.0)	32 (61.5)	0.005	0.260 (0.098~0.690)
–819C	7 (14.0)	20 (38.5)		3.839 (1.448~10.177)
–592A	43 (86.0)	32 (61.5)	0.005	0.260 (0.098~0.690)
–592C	7 (14.0)	20 (38.5)		3.839 (1.448~10.177)
Genotype, n (%)				
–1082AA	24 (96.0)	23 (88.5)	0.513	N.S.
–1082AG	1 (4.0)	2 (7.7)		N.S.
–1082GG	0 (0.0)	1 (3.8)		N.S.
–819TT	18 (72.0)	11 (42.3)	0.027	0.285 (0.089~0.918)
–819TC	7 (28.0)	10 (38.5)		N.S.
–819CC	0 (0)	5 (19.2)		N.S.
–592AA	18 (72.0)	11 (42.3)	0.027	0.285 (0.089~0.918)
–592AC	7 (28.0)	10 (38.5)		N.S.
–592CC	0 (0)	5 (19.2)		N.S.

All the *P* values represent χ^2 or Fisher’s exact test results. OR, odds ratio; CI, confidence interval. N.S., non-significant.

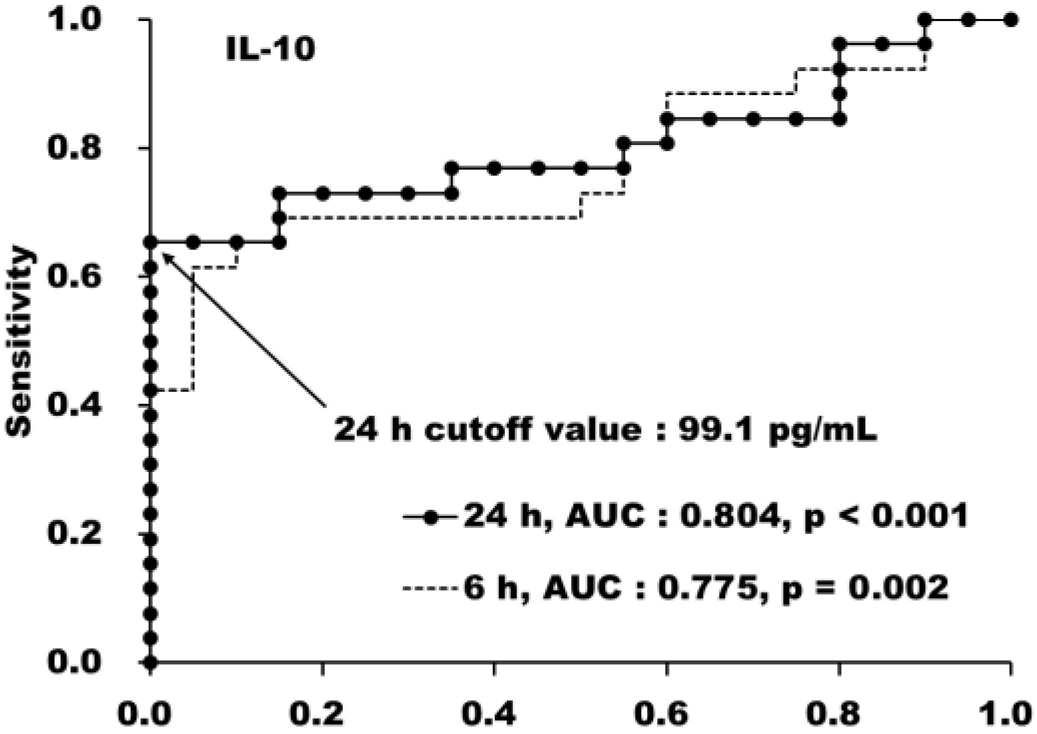

Figure S1. Receiver-operating characteristic (ROC) analysis for Interleukin-10 in predicting the outcome of 46 patients with cardiogenic shock receiving ECMO support. Plasma Interleukin-10 level measured at 24 hr after ECMO support differentiates the hospital mortality well, with the optimal cutoff value at 99.1 pg/mL.

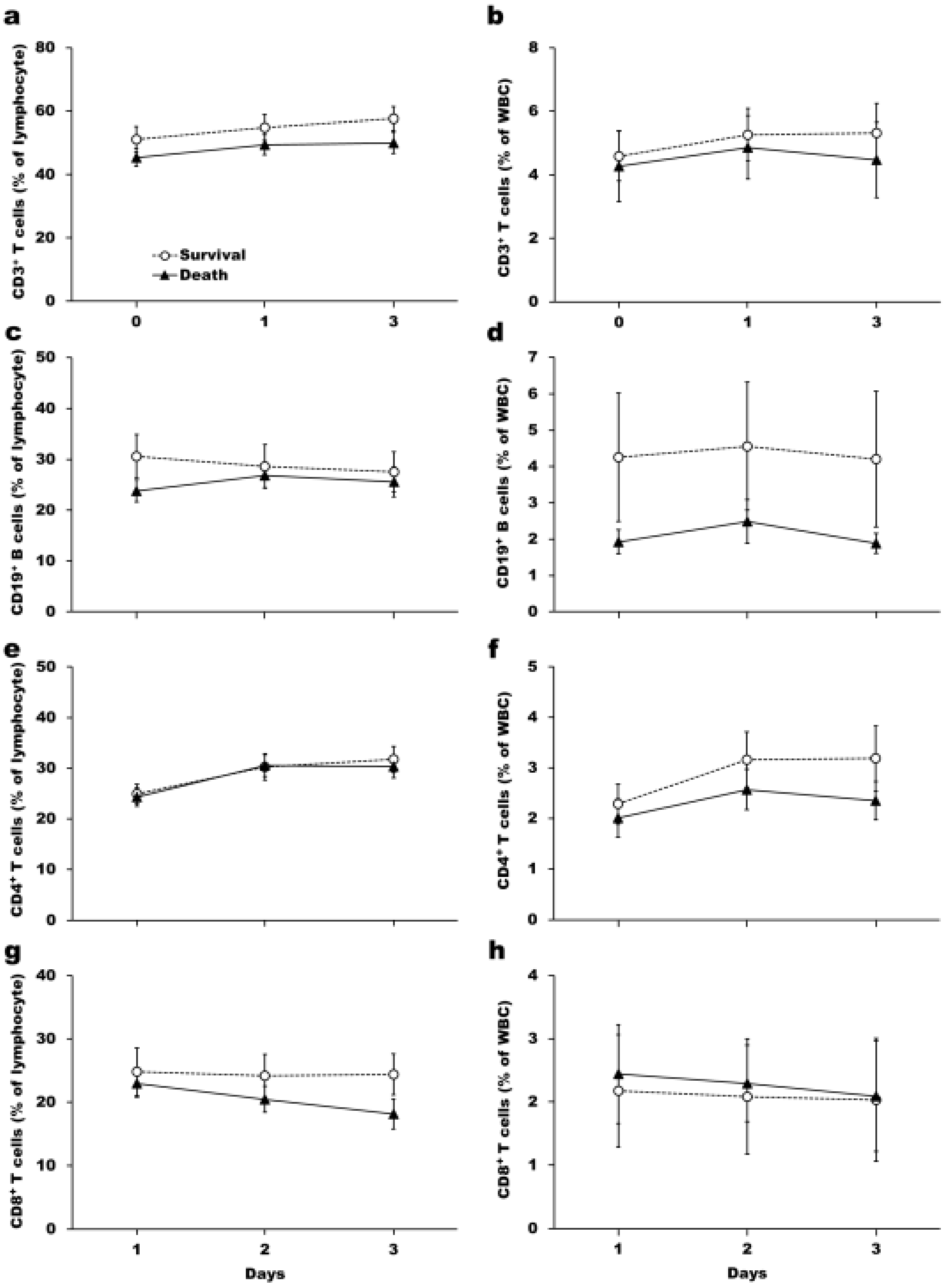

Figure S2. Comparisons of immune cell profiles between survivors and non-survivors in ARDS patients who received ECMO support. $CD3^+$ T cell percentage in lymphocytes (a), in total white blood cells (b), $CD19^+$ B cell percentage in lymphocytes (c), in total white blood cells (d), $CD4^+$ T cell percentage in lymphocyte (e), in total white blood cells (f), $CD8^+$ T cell percentage in lymphocytes (g), and in total white blood cells (h) were analyzed at day 0, 1 and 3 during ECMO support and compared between the survival and death groups. The data represented the means and standard errors of each group. Values were logarithmically transformed before bivariate comparisons.

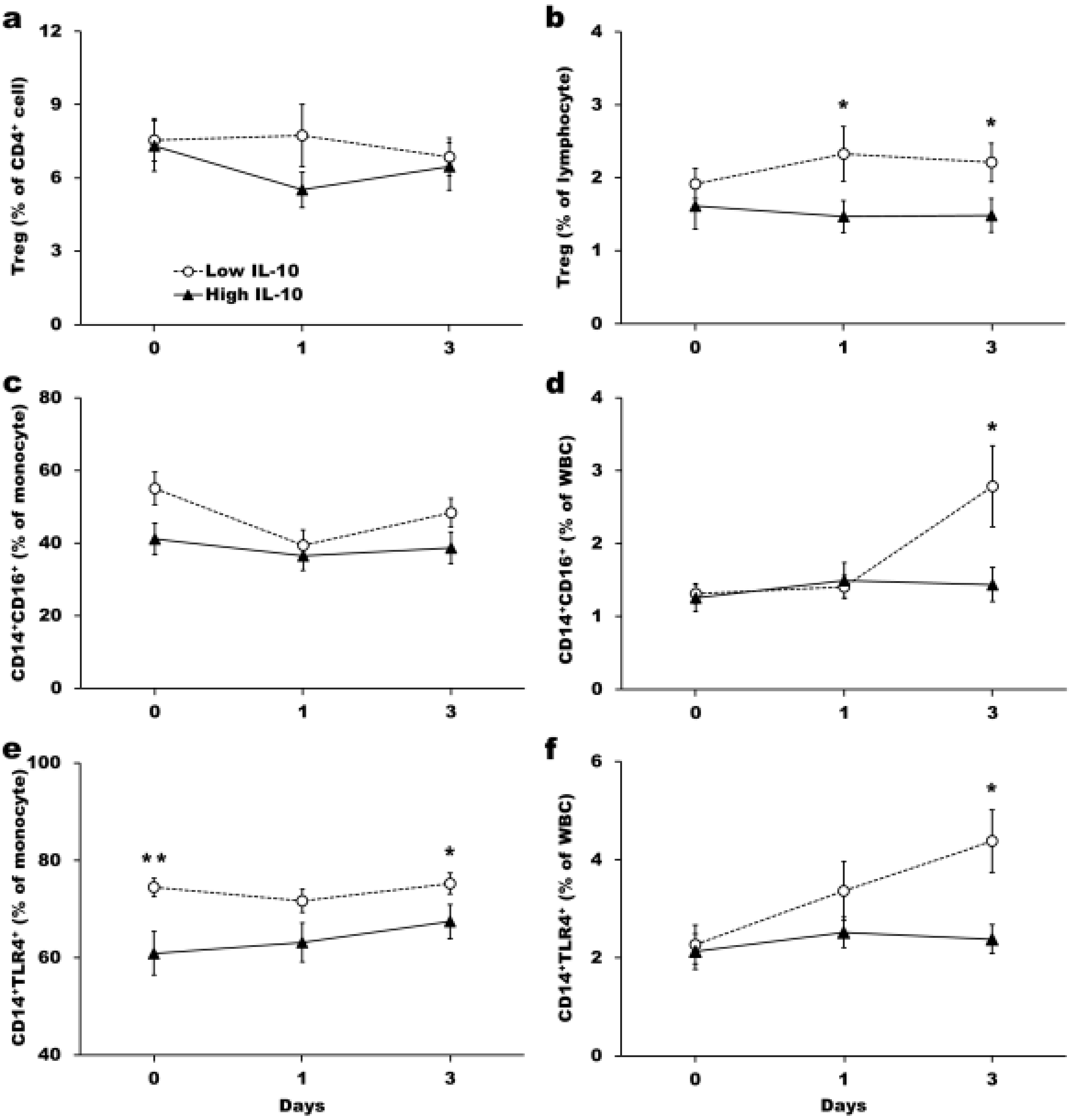

Figure S3. Comparisons of immune cell profiles according to initial IL-10 levels in ARDS patients receiving ECMO support. T regulator cell percentage in $CD4^+$ lymphocytes (a), in total lymphocytes (b), $CD14^+CD16^+$ cell percentage in monocytes (c), in total white blood cells (d), $CD14^+TLR4^+$ cell percentage in monocyte (e), and in total white blood cells (f) were analyzed at day 0, 1 and 3 during ECMO support were compared in ARDS patients according to their plasma IL-10 level at day 0. The data represented the means and standard errors of each group. Values were logarithmically transformed before bivariate comparisons. *, and ** stand for $P < 0.05$, and $P < 0.01$, respectively, between the low IL-10 (< 88.9 pg/mL) and high IL-10 (≥ 88.9 pg/mL) groups.
